# Hub gene associated with prognosis in bladder cancer is a novel therapeutic target

**DOI:** 10.7717/peerj.15670

**Published:** 2023-08-16

**Authors:** Dengpan Fang, Yuanqiao He, Yun Yi, Jiaqi Mei, Cundong Liu

**Affiliations:** 1 Department of Urology, The Third Affiliated Hospital of Southern Medical University, Guangzhou, China; 2 Department of Urology, The Sixth Hospital of Wuhan, Affiliated Hospital of Jianghan University, Wuhan, China; 3 Center of Laboratory Animal Science, Nanchang University,, Nanchang, China; 4 Jiangxi Province Key Laboratory of Laboratory Animal, Nanchang, China; 5 Nanchang Royo Biotechnology, Nanchang, China; 6 The Second Affiliated Hospital of Nanchang University, Nanchang, China; 7 The First Clinical Medical College, Nanchang University, Nanchang, China

**Keywords:** Bladder cancer, Hub gene, Prognosis

## Abstract

**Objective:**

Bladder cancer is a clinical and social conundrum due to its high incidence and recurrence rate. It is urgent to find new targets for the diagnosis and treatment of bladder cancer and improve the prognosis and survival rate of bladder cancer patients. We sought a prognosis-related gene, built related models of evaluated bladder cancer and identified the function of the hub gene in bladder cancer.

**Methods:**

We downloaded the data of bladder cancer patients from the TCGA database, and used differentially expressed genes (DEGs), copy number variation (CNV) and survival analysis to scan the hub genes associated with prognosis in bladder cancer. Then, multi-factor cox regression was used to obtain the bladder cancer prognosis correlation model. Then, we analyzed the relationship between the expression of hub gene and immune microenvironment of bladder cancer. The relationship between the expression of hub gene and prognosis in bladder cancer patients was verified by immunohistochemistry. Cell proliferation assay and drug sensitivity test *in vivo* were used to verify the inhibition of bladder cancer by targeted inhibitors.

**Results:**

In bladder cancer, we screened seven hub genes (*ACLY, CNP, NKIRAS2, P3H4, PDIA6, VPS25 and XPO1*) associated with survival. Moreover, the multifactor regression model constructed with hub gene can well distinguish the prognosis of bladder cancer. Hub gene is mostly associated with immune microenvironment. Immunohistochemical results basically confirmed the importance of XPO1 in bladder cancer. Selinexor (an inhibitor of XPO1) could effectively inhibit the proliferation of bladder cancer in the cell proliferation experiments by CCK-8 assays and it could suppress the growth of bladder cancer in mouse bladder cancer model.

**Conclusions:**

In this study, a prognostic model with seven hub genes has provided great help for the prognosis prediction of bladder cancer patients. And *XPO1* is an important target affecting the prognosis of bladder cancer, and inhibition of *XPO1* can effectively inhibit bladder cancer proliferation and growth.

## Introduction

Bladder cancer (BC), the most common malignancy of the urinary system, is one of the top ten most common cancer types in the world, with about 550,000 new cases each year, accounting for about 3% of all new cancer cases (Bray et al., 2018). Bladder cancer accounts for 2.1 percent of cancer deaths (Bray et al., 2018). Bladder cancer can be divided into non-muscle-invasive bladder cancer (NMIBC) and muscle-invasive bladder cancer (MIBC) (Knowles & Hurst, 2015). While most NMIBC patients respond well to the typical transurethral resection followed by Bacillus Calmette Guerin vaccine intravesical therapy, MIBC has proven more difficult to treat, with a five-year survival rate of about 60–70% (Patel, Oh & Galsky, 2020). Despite rapid advances in technology and medicine, including the emergence of more BC therapies such as robot-assisted techniques in surgery, molecularly targeted drugs, and immunotherapy, survival outcomes for BC patients have not improved significantly over the past 20 years (Zang et al., 2020). Therefore, how to effectively treat MIBC, improve the prognosis of MIBC patients and improve the survival rate has been an urgent clinical problem to be solved.

High-throughput sequencing technologies and construction of genomic databases such as TCGA (https://cancergenome.nih.gov/) and Gene Expression Omnibus (GEO, http://www.ncbi.nlm.nih.gov/geo/) provide lots of new information about the genomic and transcriptomic (Zhang et al., 2021). Bioinformatics and data mining have been increasingly applied to various medical researches (Zhu et al., 2022). DNA copy number variation (CNV) has long been known as a source of genetic variation, which play an important role in human disease and biology (Xie & Tammi, 2009). The CNV regions cover more nucleotide content per genome than single nucleotide polymorphisms (SNPs), which is suggesting the importance of CNV in genetic diversity (Xie & Tammi, 2009). Somatic CNV are commonly observed in cancer, which are major drivers for tumor development and drug resistance (Soong et al., 2020). There are many studies using CNV differences to analyze genetic differences in tumors (Maire et al., 2021). In RNA-seq, the expression level of each mRNA transcript is measured by the total number of mapped fragments, and RPKM (reads per kilobase of transcript per million reads mapped) was used as normalized expression units instead of integer counts directly to remove technical biases in sequenced data (Zhao, Ye & Stanton, 2020). The RPKM was used to analysis the DEGs or isoforms between two or more conditions. Multivariable Cox regression models applied to construct the best model of hub genes (Wang et al., 2021). These provide a good opportunity to study the relationship between differentially expressed genes and tumor prognosis.

This article aimed to explore the correlation between hug genes expression in bladder cancer and its correlation with clinical prognosis, which sought to establish a reliable predictive model for patients with bladder cancer. We also verified the relationship between one of the hub genes (XPO1) expression and prognosis in bladder cancer tissue specimens by immunohistochemistry (IHC). And it demonstrated that Selinexor, an inhibitor of XPO1, inhibited bladder cancer cell lines and also inhibited growth of transplanted tumor from M49 bladder cancer cell line.

## Material and Methods

### Data selection

On 23 October 2022, gene expression profiles (including 412 tumor tissues and 19 normal tissues) and copy number variation (CNV) dataset (including 417 cases of bladder cancer samples) with complete follow-up information of bladder cancer patients were downloaded from The Cancer Genome Atlas (TCGA) database (https://portal.gdc.cancer.gov/). Besides, the GSE13507 cohort from the GEO database (https://www.ncbi.nlm.nih.gov/) was used to validate the prognostic of XPO1.

### Analysis of copy number variation data

The CNV data from 417 bladder cancer samples were annotated using the Genome Research Consortium Human build 38 (GRCh38) as a reference genome. The whole-genome amplification and deletion in the sample were identified using the pipeline Genomic ldentification of Significant Targets in Cancer (GISTIC2.0) (version 2.0.23) (Mermel et al., 2011) from GenePattern (Reich et al., 2006) (https://cloud.genepattern.org/). The levels derived from GISTIC2.0 indicate the copy-number level per gene: −2 or Deep Deletion indicates a deep loss, -1 or Shallow Deletion indicates a shallow loss, 0 is diploid, 1 or Gain indicates a low-level gain, 2 or Amplification indicate a high-level amplification (Cerami et al., 2012; Gao et al., 2013).

### Identification of differentially expressed genes (DEGs)

The FPKM data of tumor and normal tissues from TCGA-BLCA were analyzed using the “limma” R-package to obtain DEGs. The threshold for DEGs was as follows: adj *P*-value (FDR)<0.01 and —log2-fold change (FC)—≥2.

### Univariate Cox regression analysis

A total of 109 chemotherapy samples were selected from 412 bladder tumor samples obtained from the TCGA-BLCA, and univariate Cox regression analysis was employed to explore the performance of DEGs in predicting overall survival (OS). Genes were determined as potential prognostic genes with *p* < 0.05. When HR ≤ 1 indicates that the gene may promote survival, HR ≥ -1 may have an adverse effect on survival. We identified genes with HR > 1 & *p* < 0.05 as risk prognostic genes.

### Construction and evaluation of prognostic model

In this study, seven risk prognostic related genes with copy number amplification and up-regulation in tumors were selected. By integrating expression profile and clinical information, we selected 406 bladder cancer samples with complete survival data, and hub genes were included in the stepwise multivariate COX regression analysis, and the riskscore was constructed according to the standardized regression coefficient of the factors: Riskscore = Σi(Coefi × Expi). Expi represents gene expression of prognostic genes, Coefi represents the gene regression coefficient in multivariate Cox regression analysis.

According to the prognostic model, we calculated the riskscores of bladder tumor samples in the TCGA database, and divided the samples into high-risk group and low-risk group based on the median value of riskscores. t-SNE was drawn by R-package “Rtsne” and the high-dimensional data combined with samples survival information and riskscore was converted into low-dimensional data and visualized to distinguish between high- and low-risk groups. By Kaplan–Meier (K–M) survival curve we identified OS between high and low risk groups. In addition, time-dependent receiver operating characteristic (t-ROC) curve analyses of patients were plotted by R packet “pROC” to assess the sensitivity of the model in predicting half-year, one-year and two-year survival of patients (Robin et al., 2011).

### Functional enrichment analysis

The Wilcoxon test was used to detect DEGs between the high- and low-risk groups. DEGs with —logFC—≥1 and FDR<0.05 were used for subsequent analysis. The R “clusterProfiler”(Yu et al., 2012) was used for the Kyoto Encyclopedia of Genes and Genomes (KEGG) enrichment (Kanehisa, 2019; Kanehisa et al., 2021; Wixon & Kell, 2000) and Gene Ontology (GO) enrichment analysis to identify DEGs-enriched pathways.

### Analysis of tumor immune microenvironment

R-package “GSVA” was used to calculate the immune score in the tumor microenvironment. We also explored the infiltration of 13 common immune cells including aDCs, B cells, CD8+ T cells, DCs, iDCs, macrophages, mast cells, neutrophils, NK cells, pDCs, T helper cells, Tfh, Th1 cells, Th2 cells, TIL, Treg. Besides, R-package “estimate” was used to calculate the immune microenvironment scores and tumor cell purity (Yoshihara et al., 2013).

### Immunohistochemistry

Samples of bladder cancer diagnosed at the Department of Pathology of The Second Affiliated Hospital of Nanchang University between September 2021 and August 2022 (the project was approved by IBR EC opinion letter of The Second Affiliated Hospital of Nanchang University) were collected and made into tissue chips. Clinicopathological data and written informed consent of patients were obtained. Automatic slicing machine was used for slicing and XPO1 (Proteintech, Rosemont, IL, USA) was dyed by automatic dyeing machine (Roche, Basel, Switzerland).

### Cell proliferation assays

The MB49, T24, and HT1376 cells were plated in a 24-well plate at 10,000 cells/well and then they were treated with Selinexor (BiochemPartner, Shanghai, China) or vehicle after cultured in an incubator for 24 h. Proliferation was assessed 24 h, 48 h, 72 h later using Cell Counting Kit-8 (CCK-8) (PointBio, Indianapolis, IN, USA). After the addition of CCK8, the cells were incubated in the incubator for 1–4 h, and then the absorbance of 450nm was measured with an enzyme marker. All conditions were performed in triplicate and repeated at least twice. Data is displayed as the mean ± standard deviation.

### Animals

All animals experiments were conducted as approved by Nanchang Royo Biotech company Laboratory Animal Welfare Ethics Committee (RYE2021072501). Six-to-eight weeks old balb/c mice were obtained from Nanchang Royo Biotech company. Mice were raised in independent ventilation cage system, and in different cages according to male and female, with 3–5 mice per cage, and the lighting cycle was day and night every day. Given enough water and chow, mice could obtain it freely. The ambient temperature is maintained at 22−25 °C and the humidity is maintained at 40–60%. Executing animal method is Euthanized with CO2 inhalation. The Experimental end point was 21 days after drug intervention or mouse tumor volume >1500 mm^3^ or necrosis of tumor ulcer in mice or mice lost more than 20% of the body weight of normal animals (the amount of tumor should be taken into account).

### Drug sensitivity test *in vivo*

Suspensions of MB49 cells (1.0*10 $\hat {}$6/200 uL) were subcutaneously injected to balb/c mice establish xenograft tumors. And when the MB49 xenograft tumors reached 50–100 mm^3^ size, the mice were randomly divided into two groups (*n* = 6), and then were treated with Selinexor 0 mg/kg/d (Control group) or 20 mg/kg (P.O; tiw/3; treatment). All the mice were treated for 2 weeks. The body weight and tumor volumes were measured every 3 days, and tumor volumes were calculated as (length × width2)/2. TGI was calculated as (1−T/C) × 100%, which T is the average tumor weight of the treatment group and C is the average tumor weight of the control group. The effect of the drug is considered as valid when TGI ≥40% and *p* < 0.05. Data were analyzed by two way ANOVA.

## Results

### The prognostic genes with CNV and expression differences were screened

**Figure 1:**
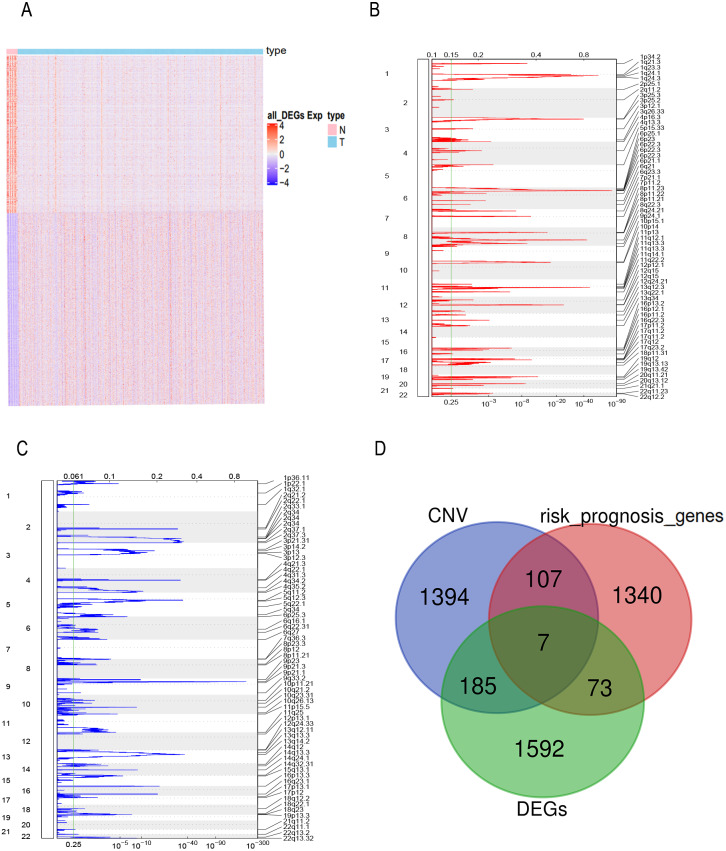
The prognostic genes with CNV and expression differences were screened. (A) mRNA expression of DEGs in bladder cancer and normal samples. Each column represents normal or tumor samples, and each row represents mRNA expression of the genes. Red indicates high gene expression, purple indicates low expression, pink and skyblue indicate normal and tumor samples, respectively. (B) Changes of gene CNV in bladder tumor samples. a plot of the G-scores (top) and q-values (bottom) with respect to amplifications for all markers over the entire region analyzed. (C) Changes of gene CNV in bladder tumor samples. a plot of the G-scores (top) and q-values (bottom) with respect to deletions for all markers over the entire region analyzed. (D) Venn diagram of upregulated DEGs, the amplified genes and risk prognostic genes.

**Figure 2:**
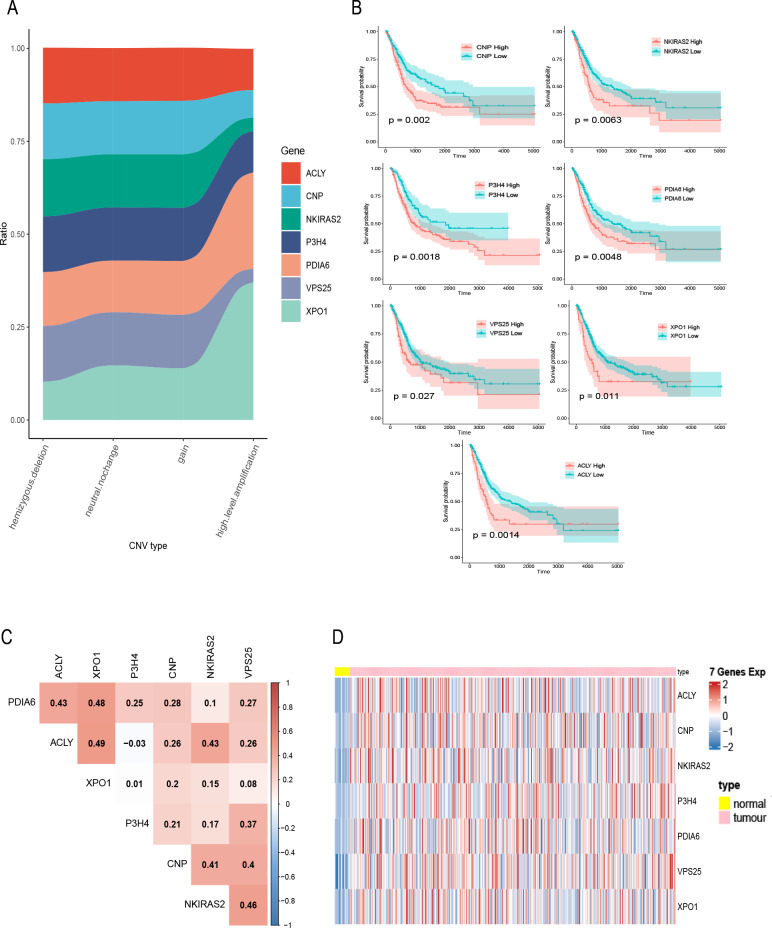
Exploration of seven prognostic genes. (A) CNV of seven genes in tumor samples, the *X*-axis represents four types of CNV and the *Y*-axis represents the proportion of the gene. (B) K–M curves of the seven genes, where the red curve indicates the survival status of the genes at high expression and the cyan curve indicates the survival status of the genes at low expression. (C) The correlation heatmap of seven genes, where numerical values indicate the correlation between two genes, red indicates a positive correlation between gene and gene, and blue indicates a negative correlation between gene module and gene. (D) mRNA expression of seven gene in bladder cancer and normal samples, each column represents normal or tumor samples, and each row represents mRNA expression of seven genes, red indicates high expression and blue indicates low expression, yellow and pink indicates normal and tumor samples, respectively.

FPKM expression profiles in TCGA-BLCA were selected as the basis for differential expression analysis. Under the condition of FDR <0.01 and —log2FC— ≥2 we identified 2,996 DEGs between bladder tumor and normal samples, including 1,857 up-regulated genes and 1,139 down-regulated genes (Fig. 1A). Through univariate Cox regression analysis, we found 80 DEGs related to the risk prognosis of bladder tumor among 1,857 up-regulated DEGs. In addition, we used GISTIC 2.0 to identify genome amplification/deletion in bladder tumor samples (Figs. 1B and 1C). Venn diagram analysis showed that seven hub genes of the 80 up-regulated DEGs (Fig. 1D) were amplified with copy number (Fig. 2A): *ACLY, CNP, NKIRAS2, P3H4, PDIA6, VPS25, XPO1.*

A total of 400 bladder tumor samples with survival data were included in TCGA database. Based on these data, the software X-tile (version 3.6.1) (Camp, Dolled-Filhart & Rimm, 2004) determined the best cut-off value of these seven hub genes and divided the samples into high-expression and low-expression groups. K–M curves of the seven hub genes all confirmed that the survival of the high expression group was worse than that of the low expression group (*P* < 0.05) (Fig. 2B). In addition, in order to explore the relationship between these seven hub genes, we mapped the correlation network containing these seven hub genes and found that their expression was positively correlated with each other (Fig. 2C). Heatmap of seven hub genes amplified and upregulated DEGs in tumor tissue are shown in Fig. 2D.

### Construction and evaluation of a prognostic model for bladder tumor

**Figure 3:**
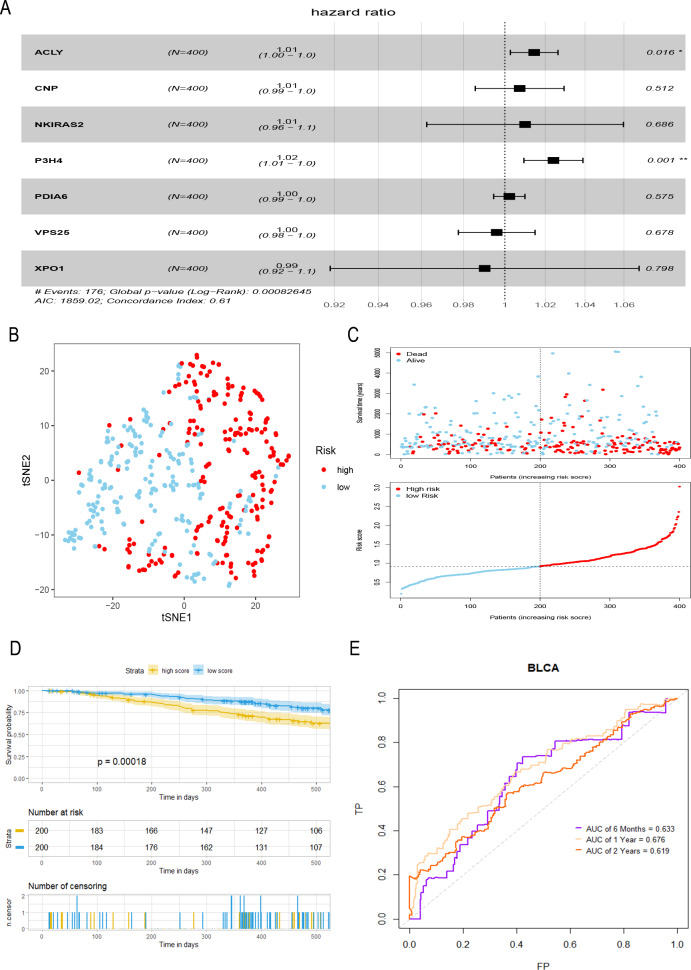
Construction and evaluation of a prognostic model for bladder tumor. (A) Multivariate Cox regression was used to construct prognostic model with seven hub genes. (B) The high- and low-risk groups were well separated by t-SNE. (C) Risk score and survival status distribution of bladder cancer. (D) K–M curves of samples in the high- and low-risk groups. (E) t-ROC curve and AUC of prognostic mode.

A total of seven hub genes were included in multivariate COX regression analysis (Fig. 3A), and a prognostic model of bladder tumor was established according to the regression coefficients: Riskscore = (0.014 *expression of *ACLY*) +(0.007 * expression of *CNP*) +(0.010 * expression of *NKIRAS2*) +(0.024 * expression of *P3H4*) +(0.002 * expression of *PDIA6*) +(−0.004* expression of *VPS25*) +(−0.010* expression of *XPO1*). After calculating the riskscore of all tumor samples, samples were divided into high- and low- risk groups with the median riskscore of 0.916. T-SNE analysis showed that high- and low-risk groups could be well distinguished (Fig. 3B). Riskplot shows that riskscore of the sample may be related to prognosis: as the patient’s riskscore increases, the patient’s survival time may decrease (Fig. 3C). The K–M curve further proved that the OS of the high-risk group was significantly worse than that of the low-risk group (*P* < 0.001) (Fig. 3D). The half-year of area under the t-ROC curve (AUC) was 0.663, 0.676 in1-year, 0.619 in 2-year (Fig. 3E).

### Gene enrichment analysis

By applying the Wilcoxon test, 219 DEGs associated with high- and low- risk groups were identified (FDR<0.05). Including 116 up-regulation genes and 103 down-regulation genes. Subsequently, KEGG and GO enrichment analysis showed that most of the high risk-related pathways were PI3K-Akt signaling pathway, laminin binding and other pathways closely related to tumor occurrence, development and metastasis (Figs. S1A–S1B).

### Analysis of the immune microenvironment

We calculated the immune score of tumor tissue using R-packet “GSVA”. After evaluating the infiltration of 13 subsets of immune cells in bladder cancer, we found that the mean proportion of T helper cells in all samples was the highest, followed by Treg (Fig. S2A). In the correlations between the expression of seven prognostic related genes and immune cells we found that that expression of *XPO1* was negatively correlated to mast cells (cor = −0.31, *p* = 2.6*e* − 10) (Fig. S2B). We further explored the correlation between the immune microenvironment, tumor cell purity and seven genes using R-packet “estimate” (Yoshihara et al., 2013). And found that except for VPS25, the other six genes were correlated with the tumor immune microenvironment. In addition, ACLY, NKIRAS2, XPO1 and tumor purity were significantly positively correlated (*p* < 0.001), which further indicates that ACLY, NKIRAS2 and XPO1 may be potential targets for the treatment of bladder cancer.

### Immunohistochemical validation of *XPO1* as a hub gene

**Figure 4:**
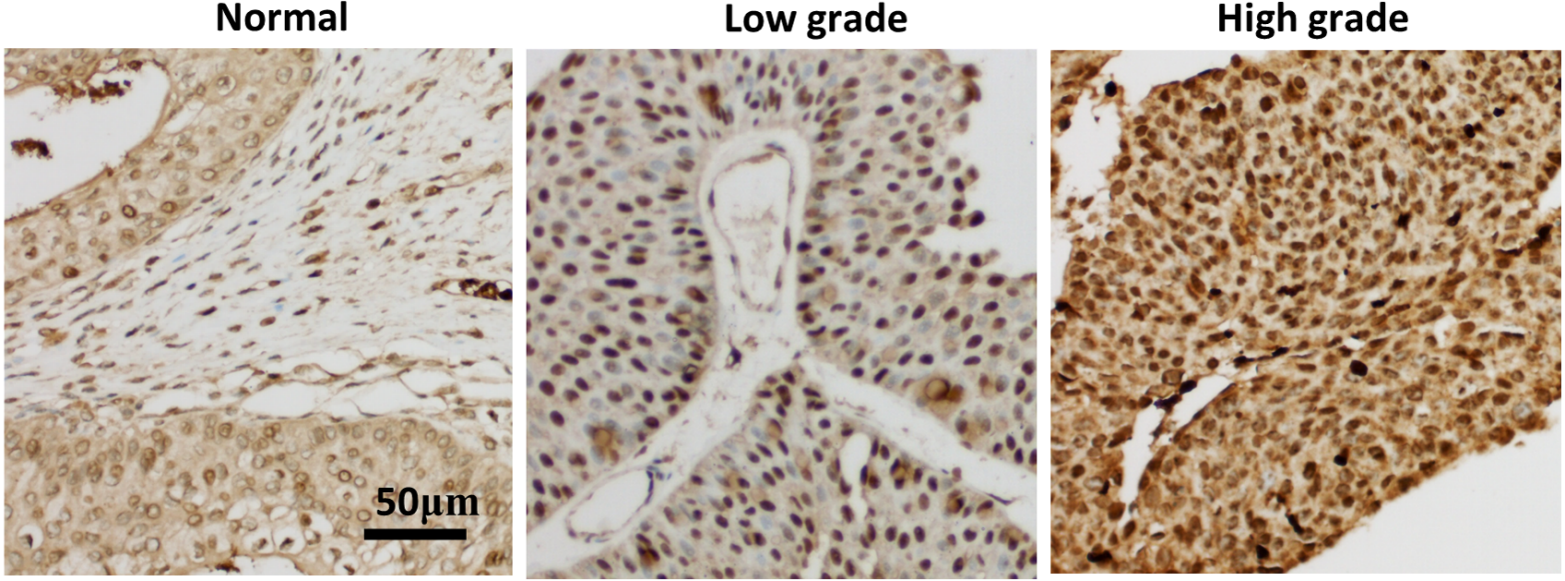
XPO1 expression was increased in both low and advanced bladder cancer. Patient’ bladder cancer tissues was immunohistochemically stained by XPO1. *n* = 3 for Normal; *n* = 7 for low grade; *n* = 8 for high grade.

In order to verify the relationship between seven hub genes and the prognosis of clinical patients with bladder cancer, we selected *XPO1* gene as the target gene to detect the expression in pathological samples of patients with bladder cancer. *XPO1* is the target of Selinexor, an antitumor drug currently on the market. Immunohistochemical analysis showed that although a small amount of immune response could be observed in the cytoplasm, *XPO1* was mainly expressed in the nucleus. The expression of *XPO1* in bladder cancer tissues was significantly higher than that in normal tissues. Moreover, the expression level of *XPO1* in advanced stages of bladder cancer was higher than that in lower stages (Fig. 4). Besides, the prognostic value of XPO1 was validated in 165 bladder cancer samples from the GSE13507 cohort of the GEO database. The best cut-off value was used to classify high and low XPO1 expression values, and the K–M curve showed that patients with high XPO1 expression had shorter survival in the external bladder cancer cohort.

### Selinexor inhibited the proliferation of bladder cancer cell lines

**Figure 5:**
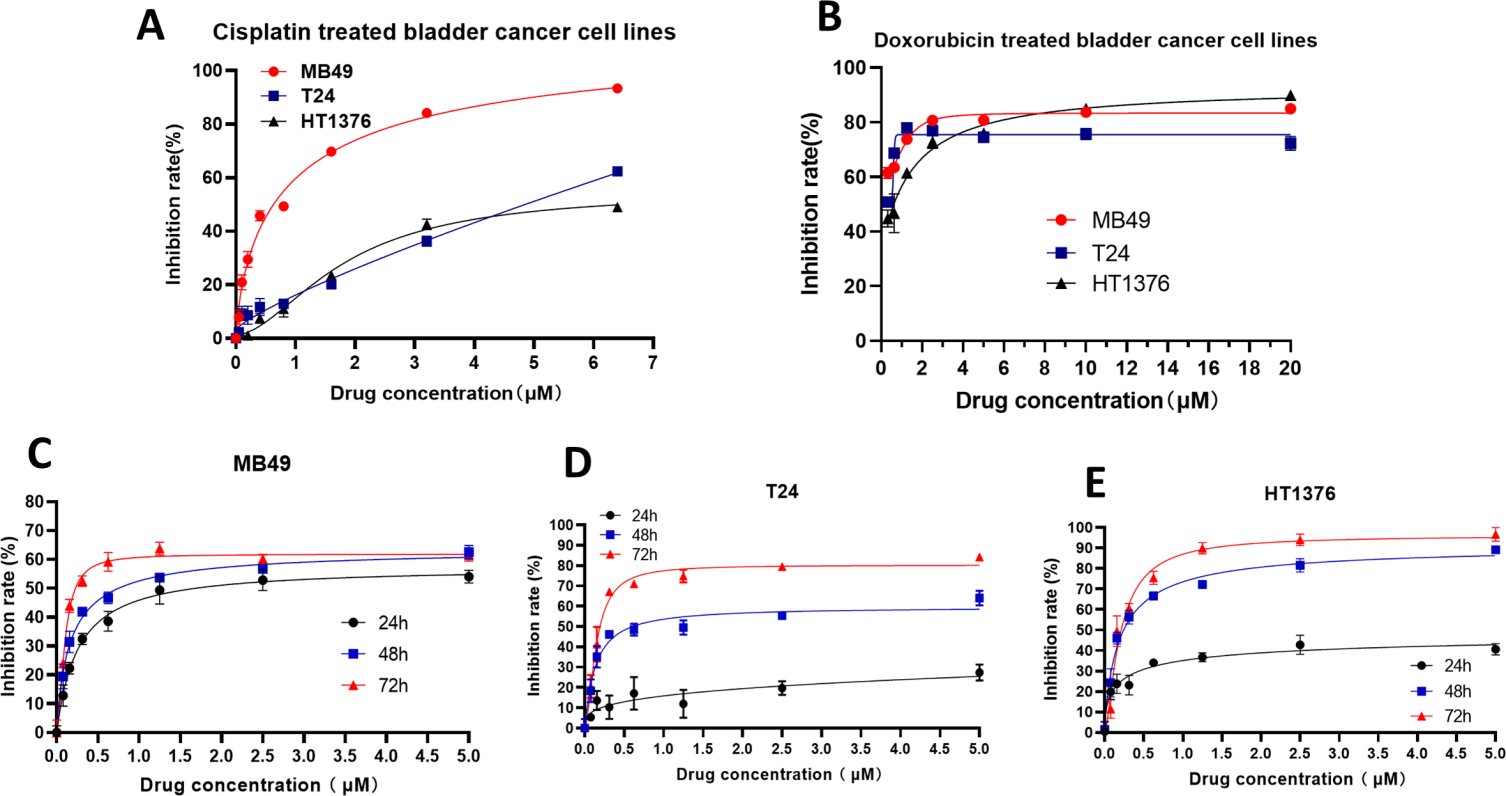
Selinexor inhibited the proliferation of bladder cancer cell lines. (A–B) Inhibitory effect of positive control drugs (cisplatin and doxorubicin) on bladder cancer cell lines. (C) Selinexor inhibited the MB49 cell line in 24 h, 28 h and 72 h, *n* = 6 per group. (D) Selinexor inhibited the T24 cell line in 24 h, 28 h and 72 h, *n* = 6 per group. (E) Selinexor inhibited the HT1376 cell line in 24 h, 28 h and 72 h, *n* = 6 per group.

To further verify the inhibitory effect of *XPO1* on bladder cancer, we examined the inhibitory effect of Selinexor, an inhibitor of *XPO1*, on murine bladder cancer cell lines MB49 and human bladder cancer cell lines T24 and HT1376. Experimental results showed that positive controls (Cisplatin and Doxorubicin) were inhibited the bladder cancer lines (Figs. 5A–5B), and the IC50 of Selinexor against MB49 cells was 1.483 (in 24 h), 0.8886 (in 48 h), and 0.4408 (in 72 h) (Fig. 5C). The maximum inhibition rate of Selinexor on T24 and HT1376 cell lines was less than 50% in 24 h. IC50 of Selinexor on T24 cells was 0.8553 (in 48 h), and 0.2467 (in 72 h) (Fig. 5D); The IC50 of Selinexor on HT1376 cells was 0.2702 (in 48 h), and 0.2138 (in 72 h) (Fig. 5E). These results indicated that Selinexor could significantly inhibit the growth of MB49 and T24 bladder cancer cells.

### Selinexor inhibited the growth of bladder cancer grafts *in vivo*

**Figure 6:**
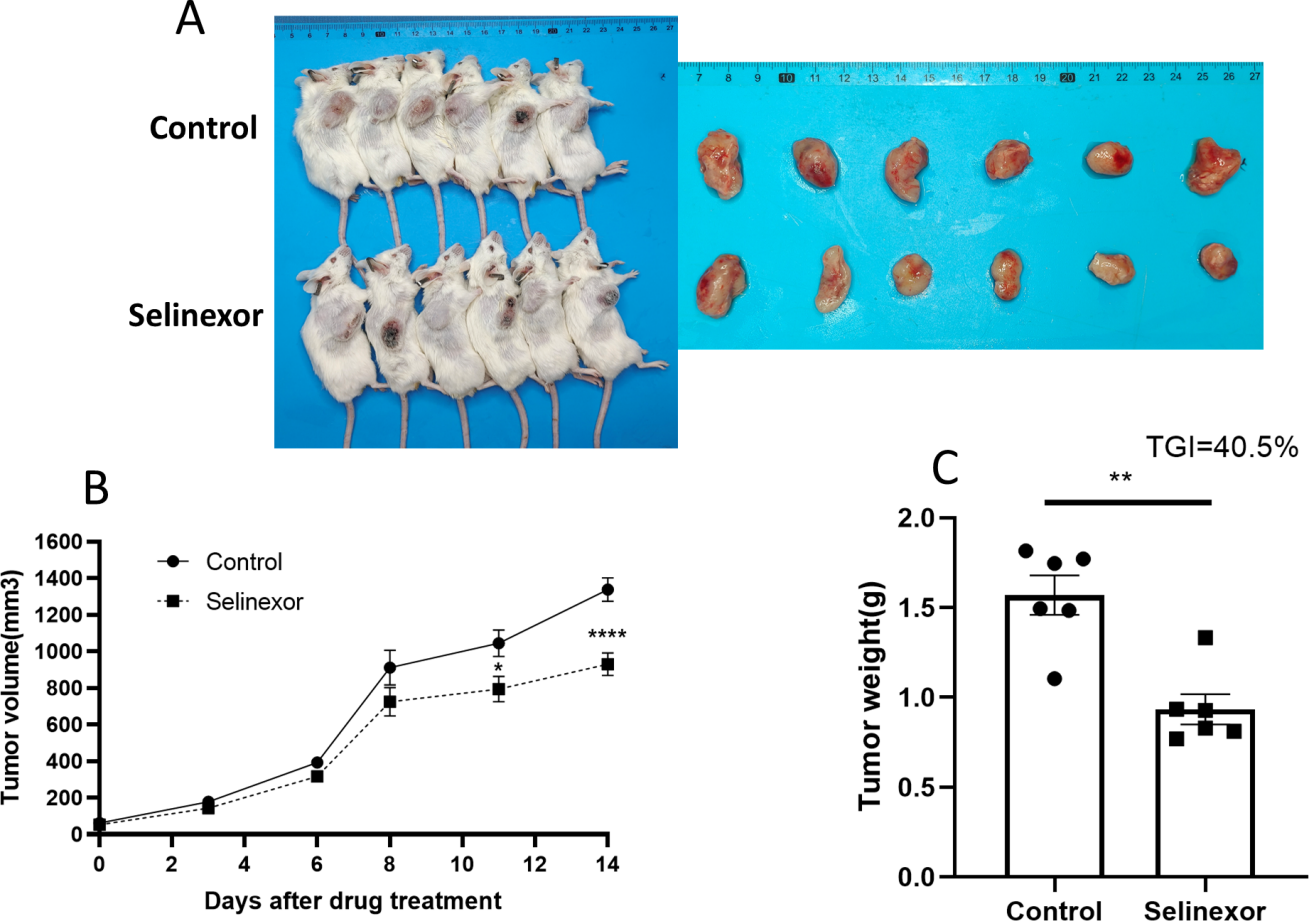
Selinexor inhibits the growth of bladder cancer grafts *in vivo*. (A) General and tumor photograph of model. (B–C) Statistical data showed that Selinexor inhibited the MB49 tumor volume, tumor weight, and TGI, *n* = 6 per group, *p < 0.05; **p < 0.01; ****p < 0.0001.

Next, we tested the *in vivo* effects of Selinexor on bladder cancer. We successfully constructed MB49 bladder cancer grafts. The mice of MB49 bladder cancer grafts were treated by Selinexor for 2 weeks. The results showed that the growth of bladder cancer grafts was significantly inhibited in the twelfth, fifteenth and eighteenth days of Selinexor treatment (Fig. 6). The TGI in Selinexor was 40.5% (much greater than 30%). That confirmed Selinexor significantly inhibited the bladder cancer grafts *in vivo*.

## Discussion

Although BC patients have more and more treatment options, including molecular targeted drugs (Conforti et al., 2015), neoadjuvant chemotherapy (Von der Maase et al., 2005), immunotherapy (Goodspeed, Jean & Costello, 2019), etc., the main treatment methods of BC have not changed significantly. In many parts of the world, the incidence and mortality rate of BC are still on the rise, which is still a considerable public health challenge. We obtained seven prognostic related bladder cancer genes by bioinformatics method, and established the prognostic related model of bladder cancer, which has great reference value for the treatment of bladder cancer.

The genesis and development of tumor is a complex process, which is regulated by many genes (Wang et al., 2021). There are many factors affecting the prognosis of tumor (Liu et al., 2022). It would be a more reliable method to evaluate the prognosis of tumor with multiple genes. Our result showed that poor prognosis in bladder cancer is associated with overexpression of *ACLY, CNP, NKIRAS2, P3H4, PDIA6, VPS25 and XPO1.* The expression of these seven genes was positively correlated. It will be a more accurate method to construct a prognostic model of bladder cancer with these seven hub genes. ATP citrate lyase (*ACLY*) is a cytosolic homotetrameric enzyme that catalyzes the conversion of citrate and coenzyme A (CoA) to acetyl-CoA and oxaloacetate, with the simultaneous hydrolysis of ATP to ADP and phosphate. *ACLY* expression and activity proved to be aberrantly expressed in liver cancer, colorectal cancer, lung cancer, prostate cancer, bladder cancer, stomach cancer and other cancers (Granchi, 2018; Merino Salvador et al., 2017; Wen et al., 2019), and its pharmacological or genetic inhibition significantly inhibited cancer cell proliferation and induced apoptosis. It was reported *ACLY* promoted glycolysis through the enhancement of the activities of PFK 1 and 2 with concomitant activation of oncogenic drivers such as PI3K/AKT (Icard et al., 2020). C-type natriuretic peptide (*CNP*) is an anti-proliferative peptide produced mainly in the vasculature and in the nervous system, which is a new potential marker in human prostate cancer (Lippert et al., 2015). NF- *κ*B inhibitor interacting Ras-like 2(*NKIRAS2*), a negative regulator of the NF- *κ*B signaling, was identified to be a direct target of miR-BART13 in nasopharyngeal carcinoma (NPC) cells, and promoted nasopharyngeal carcinoma cell growth (Xu et al., 2019). It reported that *P3H4* was highly expressed in BC tissues, and knockdown *P3H4* expression not only inhibited BC cell proliferation, cell cycle, migration and invasion, and also inhibited BC growth (Hao et al., 2020). It was confirmed that *P3H4* expression associates with poor prognosis in bladder cancer (Zhang et al., 2022). Previous studies have shown that *PDIA6* is overexpressed in BC tissues and cell lines. In addition, down-regulation of *PDIA6* significantly inhibited the proliferation and invasion of BC, and reduced BC tumor volume, weight and metastasis (Cheng et al., 2017). *VPS25* was upregulated in glioma tissues, and it was correlated with poor prognosis in glioma patients (Zhu et al., 2021). *XPO1* is the most important nuclear export transporter involved in the transport of tumor suppressor and growth regulator proteins. *XPO1* is overexpressed in many tumors, and this overexpression is associated with disease progression and lower overall survival.

Our results found that seven hub genes were associated with most immune cells, except for the *VPS25* gene. Some of these genes are positively correlated with immunity (*e.g.*, *ACLY, NKIRAS2*), while others are negatively correlated with immunity (*e.g.*, *CNP, P3H4, PDIA6*). The immune system plays a crucial role in the development of cancer (Pio et al., 2019). The cancer immune cycle is defined as a series of functional stage events that effectively control cancer growth by the immune system (Cao & Yan, 2020). Clinical tumors are often caused by the failure of immune surveillance in patients (Gao et al., 2013). With the development of tumor, tumor immune tolerance is induced under the action of various factors, tumor cells eventually showed malignant proliferation, invasion, metastasis and drug resistance (Liu et al., 2022). Therefore, many genes may influence tumor prognosis by influencing immune system effects.

Our results are consistent with previous reports that *XPO1* is weakly expressed in normal bladder tissues and highly expressed in bladder cancer (Baek et al., 2018). Our result showed that showed that the weak expression of *XPO1* was mainly concentrated in the bladder transitional epithelial cells. The inhibitory effect of Selinexor on a variety of human bladder cancer cell lines (T24, TCCSUP, J82, UM-UC-3) has been reported in the literature (Baek et al., 2018), but the effect of Selinexior on murine bladder cancer cell lines and transplanted tumors is unknown. Considering the relationship between *XPO1* and tumor immunity, we selected not only several human bladder cancer cell lines (T24, HT1376), but also murine cell line MB49 as experimental materials. We tested the effect of Selinexor on the MB49 transplanted tumors with the normal immunity mice. Our results showed that Selinexor significantly inhibited MB49 cell lines in the normal immunity mice. We speculate that Selinexor has a direct killing effect on bladder cancer, and may also induce the corresponding anticancer effect by modulating the immune system.

## Conclusion

We have seven hub genes associated with bladder cancer prognosis. These seven hub genes play an important role in the prognosis of bladder cancer, and a prognostic model of bladder cancer was successfully constructed. At the same time, we verified that one of hub genes *XPO1* has an important effect on the treatment of bladder cancer.

##  Supplemental Information

10.7717/peerj.15670/supp-1 Supplemental Information 1 Author checklist

10.7717/peerj.15670/supp-2 Supplemental Information 2 Supplementary Figures

10.7717/peerj.15670/supp-3 Supplemental Information 3 Fig 7 raw data The inhibition of Selinexor in bladder cancer lines.

10.7717/peerj.15670/supp-4 Supplemental Information 4 Fig 8 raw data The growth of Bladder cancer treated by Selinexor.

## Additional Information and Declarations

### Data Availability

The following information was supplied regarding data availability: The raw measurements are available at figshare: He, Yuanqiao (2023). Hub gene associated with prognosis in bladder cancer is the noval therapeutic target. figshare. Dataset. https://doi.org/10.6084/m9.figshare.22273258.v3.
