# Supplementary material for: Hub gene associated with prognosis in bladder cancer is a novel therapeutic target
Source: PeerJ. 2023 Aug 16;11:e15670. doi: 10.7717/peerj.15670 (PMC10439716; doi:10.7717/peerj.15670)
Supplement: Supplemental Information 2 [file peerj-11-15670-s002.docx]

**Supplementary**

**Hub gene associated with prognosis in bladder cancer is the noval therapeutic target**

Dengpan Fang^1,2^, Yuanqiao He^3,4,5^, Yun Yi^6^, Jiaqi Mei^7^, Cundong Liu^1^*

^1^ Department of Urology, The Third Affiliated Hospital of Southern Medical University, Guangzhou, Guangdong, China

^2^ Department of Urology, The Sixth Hospital of Wuhan, Affiliated Hospital of Jianghan University, Wuhan, Hubei, China

^3^ Center of Laboratory Animal Science, Nanchang University, Nanchang, Jiangxi, China

^4^ Jiangxi Province Key Laboratory of Laboratory Animal, Nanchang, Jiangxi, China

^5^ Nanchang Royo Biotechnology, Nanchang, Jiangxi, China

^6^ Biobank center, The Second Affiliated Hospital of Nanchang University, Nanchang, Jiangxi, China

^7^ The First Clinical Medical College, Nanchang University，Nanchang, Jiangxi, China

* Corresponding author:

Cundong Liu;

Address: No. 183, Zhongshan Avenue West, Tianhe District, Guangzhou, 510630, Guangdong Province, China

E-mail: [cundongliu@163.com](mailto:cundongliu@163.com);


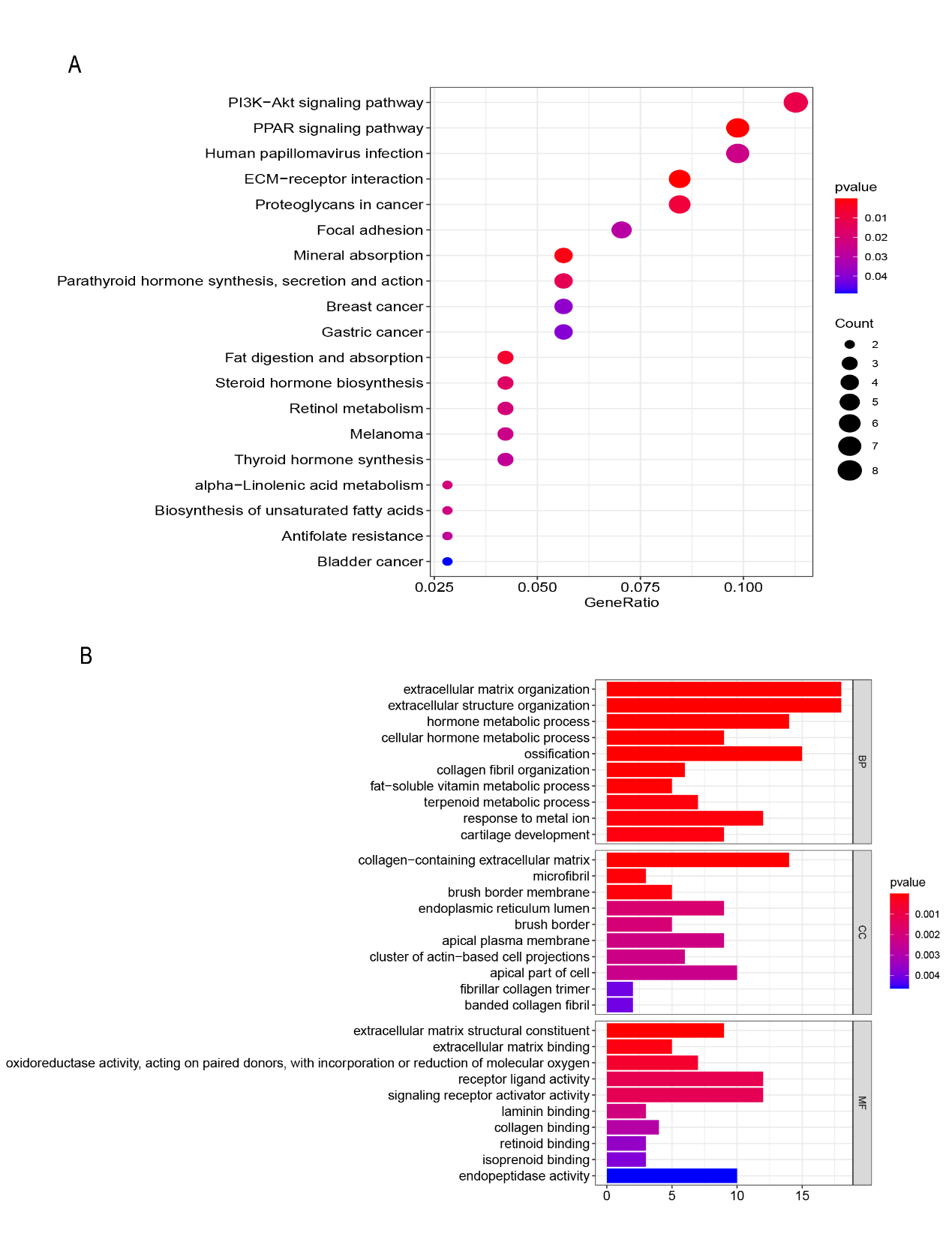


Fig. S1A: KEGG enrichment analysis in high-risk and low-risk groups; S1B: GO enrichment analysis in high-risk and low-risk groups


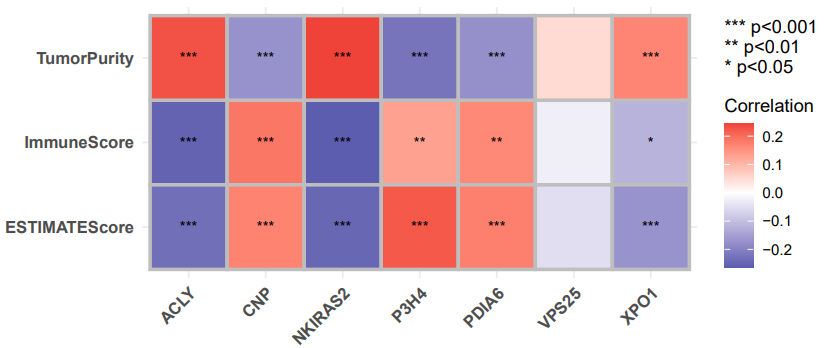

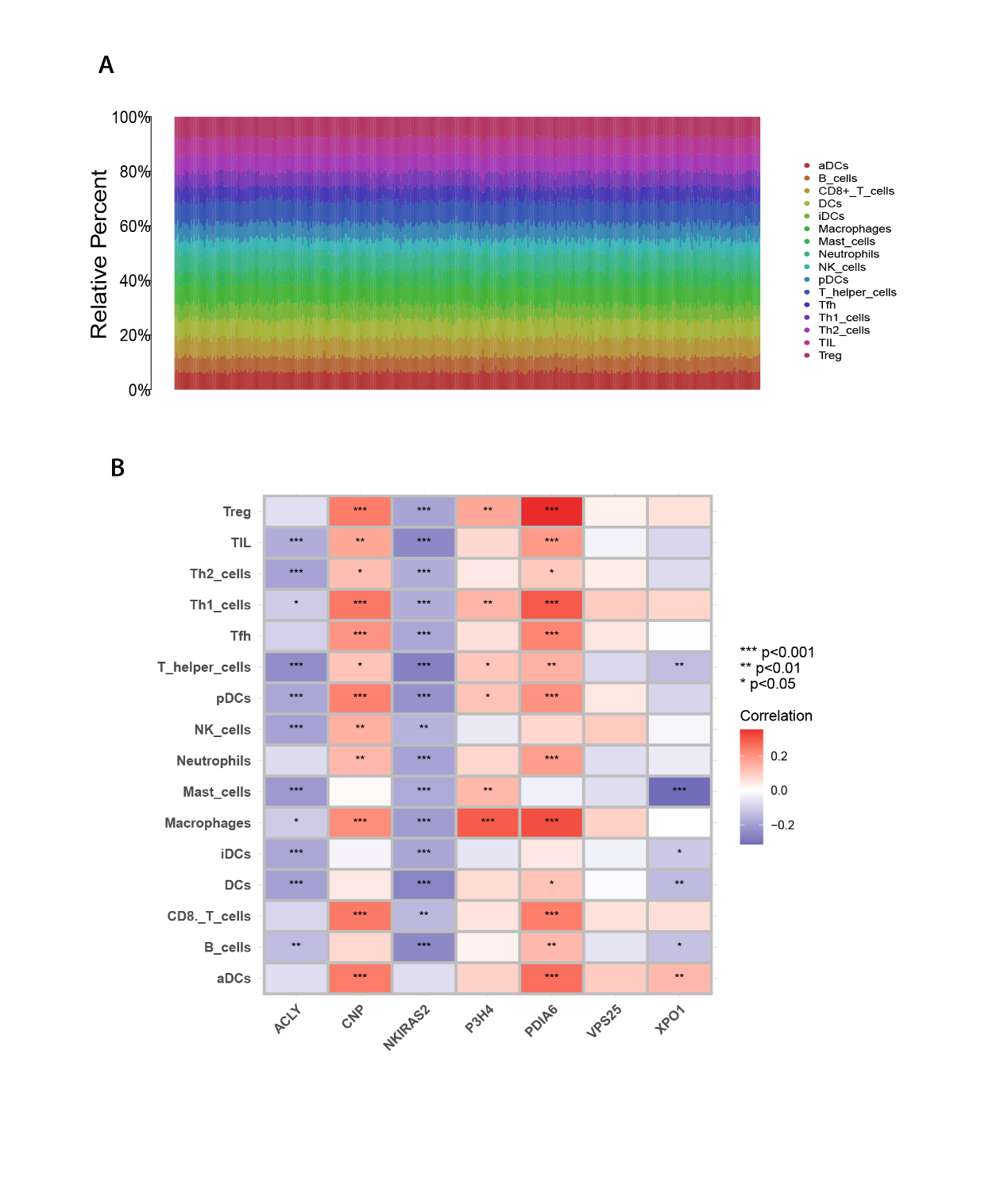


**C**

Fig. S2A: The fraction of 13 subsets of immune cells in bladder cancer; S2B: Correlation between 13 immune cells and 7 genes; S2C: Correlation between immune microenvironment and tumor purity and genes
